# Residues His172 and Lys238 are Essential for the Catalytic Activity of the Maleylacetate Reductase from *Sphingobium chlorophenolicum* Strain L-1

**DOI:** 10.1038/s41598-017-18475-8

**Published:** 2017-12-22

**Authors:** Lifeng Chen, Ed S. Krol, Meena K. Sakharkar, Haseeb A. Khan, Abdullah S. Alhomida, Jian Yang

**Affiliations:** 1 College of Pharmacy and Nutrition, University of Saskatchewan, 107 Wiggins Road, Saskatoon, SK S7N 5E5 Canada; 2 Department of Biochemistry, College of Science, King Saud University, Riyadh, Saudi Arabia; 3 Present Address: Agrisoma Biosciences Inc., 4410-110 Gymnasium Place, Saskatoon, SK S7N 0W9 Canada

## Abstract

Maleylacetate reductase (PcpE), the last enzyme in the pentachlorophenol biodegradation pathway in *Sphingobium chlorophenolicum* L-1, catalyzes two consecutive reductive reactions, reductive dehalogenation of 2-chloromaleylacetate (2-CMA) to maleylacetate (MA) and subsequent reduction of MA to 3-oxoadipate (3-OXO). In each reaction, one molecule of NADH is consumed. To better understand its catalytic function, we undertook a structural model-based site-directed mutagenesis and steady-state kinetics study of PcpE. Our results showed that the putative catalytic site of PcpE is located in a positively charged solvent channel at the interface of the two domains and the binding of 2-CMA/MA involves seven basic amino acids, His172, His236, His237, His241 and His251, Lys140 and Lys238. Mutagenesis studies showed that His172 and Lys238 are essential for the catalytic activity of PcpE. However, the mutation of His236 to an alanine can increase the catalytic efficiency (*k*_*cat*_/*K*_*m*_) of PcpE by more than 2-fold, implying that PcpE is still in an early stage of molecular evolution. Similar to tetrachlorobenzoquinone reductase (PcpD), PcpE is also inhibited by pentachlorophenol in a concentration-dependent manner. Furthermore, our studies showed that PcpE exhibits an extremely low but detectable level of alcohol dehalogenase activity toward ethanol and supports the notion that it is evolved from an iron-containing alcohol dehydrogenase.

## Introduction

Pentachlorophenol (PCP) is a potent synthetic uncoupler of oxidative phosphorylation. It was widely used as a low-cost and effective pesticide in the agriculture and timber industries in the last century^1–3^. In the 1970s and 1980s however, PCP was observed to be highly toxic to fish, farm animals and humans^4–6^. Extensive exposure to PCP has been associated with neurological disorders, acute renal failure, endocrine disorders, repeated miscarriages, pancreatitis, and cancer^7–11^. In 1987, the United States Environmental Protection Agency banned the commercial usage of PCP^12^. Unfortunately, PCP is highly resistant to bacterial biodegradation due to the presence of high and obstructive halogenation in its chemical structure^13^. Persistence of PCP in soils and waters imposes a continuous source of contamination to agricultural products such as grains, vegetables and fruits, which contributes to 99.9% of the total PCP intake in the United States^14^. Currently, PCP is listed as one of the major environmental pollutants in North America.

During the past three decades, a number of soil and aquatic bacteria have been discovered to possess the capability of degrading PCP and using its ring-cleavage products as their sole carbon source^15–19^. In spite of low catalytic activities for the enzymes in the PCP biodegradation pathways, these bacteria provide a possible bioremediation strategy for environmental PCP contaminations. Among these bacteria, *S. chlorophenolicum* L-1 is the most efficient and most studied bacterium. Its PCP-biodegradation pathway consists of six catalytic enzymes (PcpA, PcpB, PcpC, PcpD, PcpE and PcpF) encoded by genes distributed in two DNA fragments^20–26^. Furthermore, two potential LysR-type transcriptional regulators (PcpR and PcpM) were identified to regulate the expression of the catalytic enzymes^25,27,28^. PcpB (pentachlorophenol 4-monooxygenase) is the first and rate-limiting enzyme in the PCP-biodegradation pathway in *S. chlorophenolicum* L-1. Maleylacetate reductase (PcpE), which belongs to the iron-containing alcohol dehydrogenase superfamily, is the last catalytic enzyme in the pathway^25,26^.

**Figure 1:**
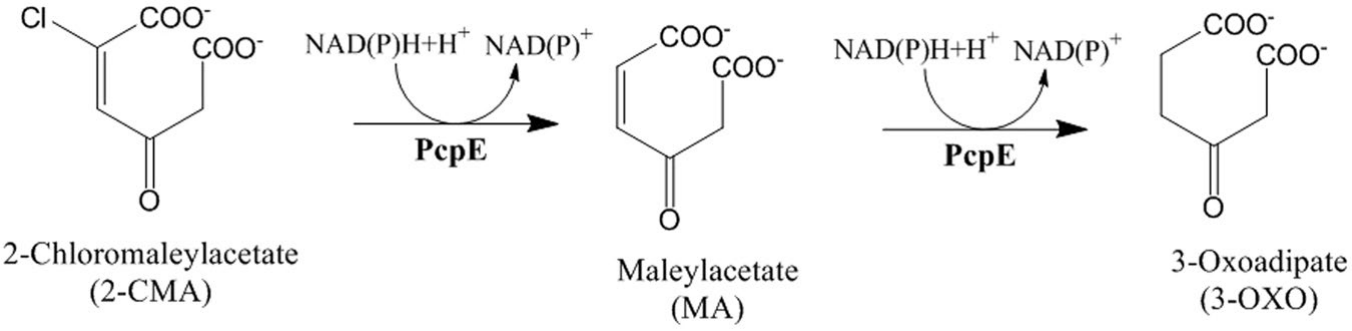
The consecutive reductive reactions catalyzed by maleylacetate reductase (PcpE) from *S. chlorophenolicum* L-1.

PcpE, which contains 352 amino acid residues, is encoded by a single copy of the gene *pcpE* located in the *pcpEMAC* fragment^25^. Its expression requires induction by PCP and regulation by the transcriptional regulator PcpR^25^. Mutation of *pcpR* decreased the expression of *pcpE* ^25^. PcpE catalyzes the reductive dehalogenation of 2-chloromaleylacetate (2-CMA) to maleylacetate (MA) and the subsequent reduction of MA to 3-oxoadipate (3-OXO) using either NADH or NADPH as the co-substrate [Fig. 1]^25,26^. Our previous study showed that PcpE reaches its maximal NADH-dependent catalytic activity at pH 7.0 against MA, with apparent *k*_*cat*_ and *K*_*m*_ measured at 1.2 ± 0.3 s^−1^ and 0.09 ± 0.04 mM, respectively^26^. In the current research, we undertook studies on PcpE under three aspects in order to better understand its biological functions. Firstly, we measured the kinetic parameters of PcpE towards its substrates 2-CMA and MA, and identified that two amino acid residues, His172 and Lys238, are essential for the catalytic activity of PcpE using a structural model-based site-directed mutagenesis strategy. Secondly, we investigated whether the catalytic activity of PcpE could be affected in the presence of PCP, as PCP may accumulate to higher concentrations within the *S. chlorophenolicum* L-1 cells due to extremely low catalytic efficacy of the rate-limiting enzyme PcpB^21,23^. Finally, we examined whether PcpE, an enzyme evolved from an iron-containing alcohol dehydrogenase, possesses any alcohol dehydrogenase activity towards ethanol, since the PCP-biodegradation pathway in *S. chlorophenolicum* L-1 was assembled over a very short period of time and is still in the early stage of molecular evolution.

## Results and Discussion

### Structural model of PcpE

**Figure 2:**
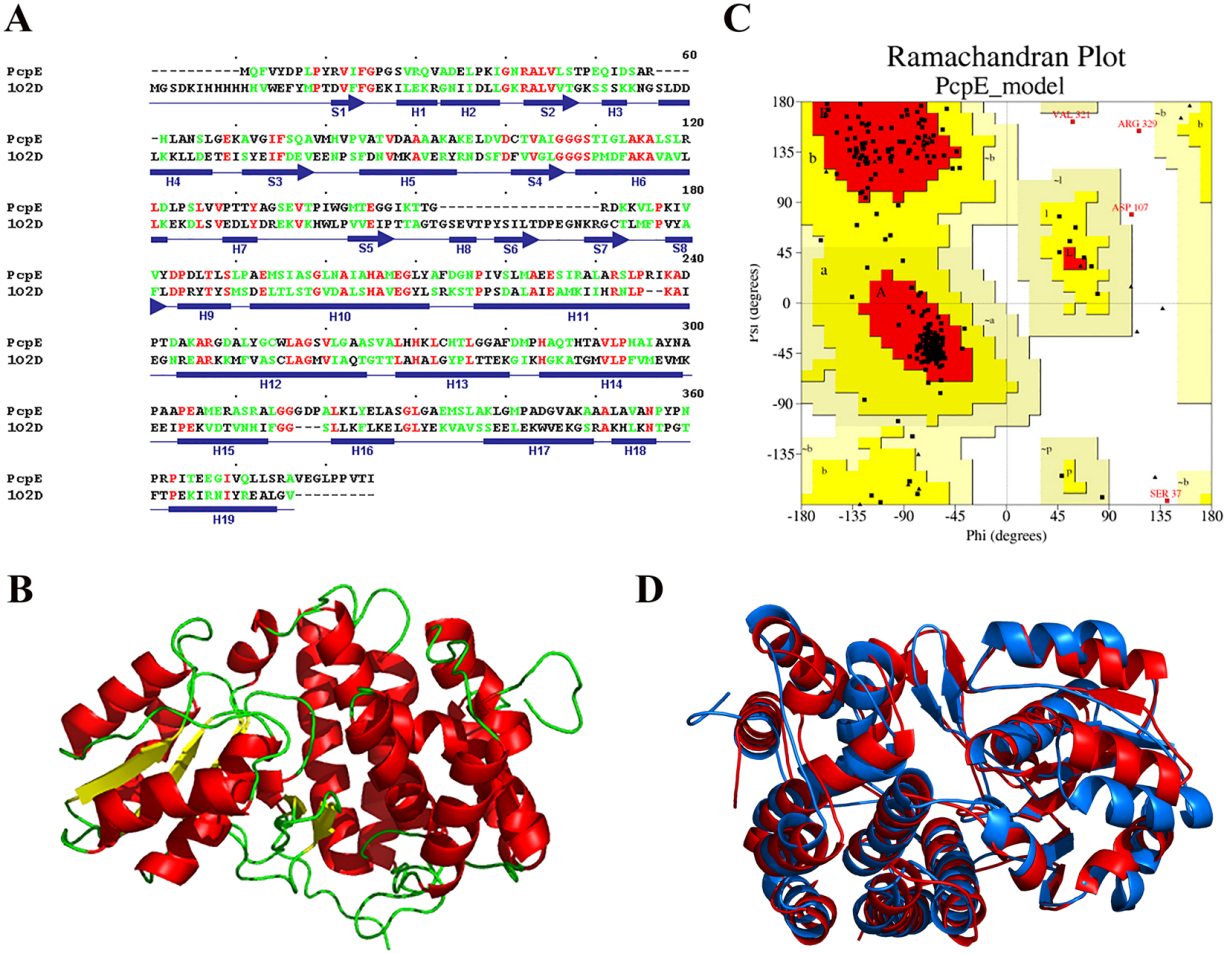
Structural homology/comparative modeling of PcpE. (**A**) Pairwise sequence alignment between PcpE and the iron-containing alcohol dehydrogenase from *Thermotoga maritima* (PDB ID code: 1O2D) with identical residues shown in red and homologous residues shown in green. The secondary structure of 1O2D was also presented with the β–strands shown in arrows and the α–helices shown in rectangles. (**B**) A ribbon representation of the structural model of PcpE. (**C**) Ramachandran plot of the structural model of PcpE with 95% of the amino acid residues in the most-favored regions. (**D**) Superimposition of the structural model of PcpE with the crystal structure of the maleylacetate reductase from *Rhizobium* sp. strain MTP-10005 (*Rm*-MA) with PcpE shown in blue and Rm-MA shown in red. The RMSD is 1.7 Å for the C_α_ carbons between the PcpE model and the *Rm*-MR crystal structure.

The catalytic behavior of an enzyme is mainly decided by its three-dimensional structure. At the time we initiated this study, no crystal structure of any maleylacetate reductase had been determined. Thus, we decided to build a three-dimensional structural model of PcpE using protein homology/comparative modeling technique. The iron-containing alcohol dehydrogenase from *Thermotoga maritima* (*Tm*-ADH, PDB ID code: 1O2D) was selected as the model template^29^. Pairwise sequence alignment showed that PcpE shares 21% sequence identity and 36% sequence homology with *Tm*-ADH [Fig. 2(A)]. The structural model of PcpE consists of two domains (an N-terminal α/β domain and a C-terminal α–helical domain) and contains seven β-strands and eighteen α-helices (including 3_10_-helices) [Fig. 2(B)], with 95% of the amino acid residues within the most-favored regions in the Ramachandran plot [Fig. 2(C)]. The contents of β-strand and α-helix in the structural model are 12.5% and 56.0%, respectively. Recently, the crystal structure of the maleylacetate reductase from *Rhizobium* sp. strain MTP-10005 (*Rm*-MR, PDB ID code: 3W5S), which shares 52% sequence identity with PcpE, was determined^30^. Retrospectively, we performed a comparison study of the PcpE model and the *Rm*-MR structure to further validate the quality of our homology/comparative modeling. As shown in Fig. 2(D), the superimposition showed that the PcpE model and the *Rm*-MR structure possess almost identical secondary structures and overall folding. The RMSD (root-mean-square deviation) is 1.7 Å for the C_α_ carbons between the PcpE model and the *Rm*-MR crystal structure. Thus, this retrospective study demonstrated that the PcpE model is highly likely to be accurate and valid to guide our structural model-based mutagenesis and kinetics studies.

### Putative 2-CMA/MA binding site

**Figure 3:**
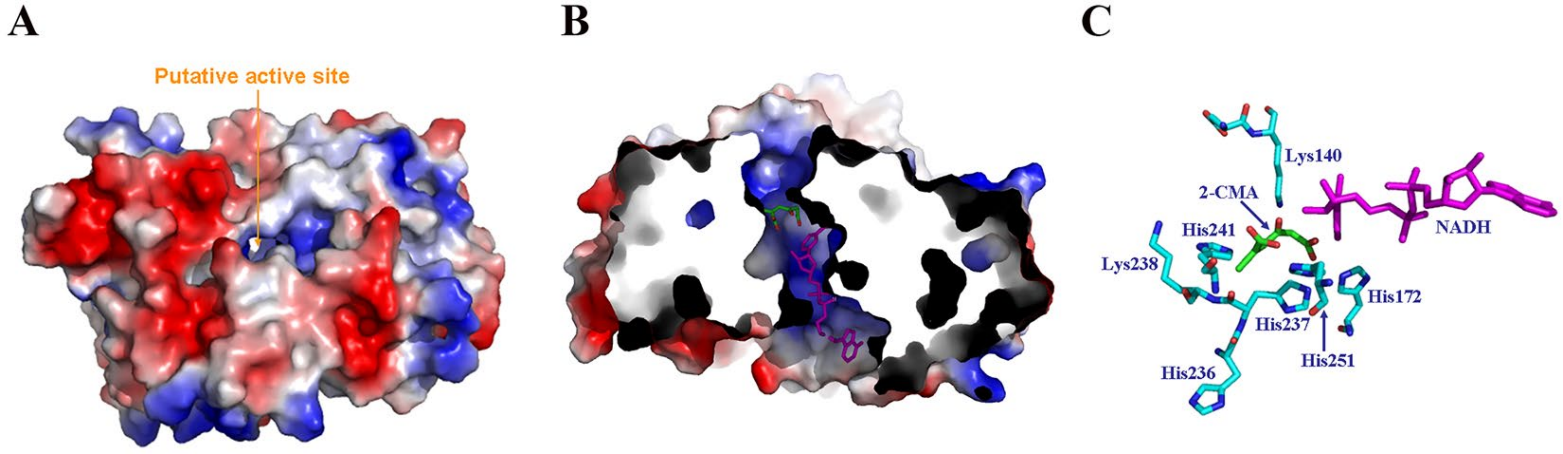
Docking of substrates 2-CMA and NADH to the putative catalytic site of PcpE. (**A**) Representation of the electrostatic potential distribution of the PcpE model with the putative active site located in a positive-charged solvent channel. (**B**) Representation of the docked 2-CMA (shown in green and red) and NADH (shown in magenta) at the positive-charged putative catalytic site. (**C**) Putative binding site of 2-CMA formed by seven basic amino acid residues, His172, His236, His237, His241 and His251, Lys140 and Lys238.

PcpE consists of an N-terminal α/β domain and a C-terminal α–helical domain with the catalytic site located at the interface of the two domains. An electrostatic potential calculation of the PcpE model showed that the putative catalytic site was located within a positively charged solvent channel [Fig. 3(A)]. We docked both substrate 2-CMA and co-substrate NADH into the putative catalytic site [Fig. 3(B)]. A cluster of seven basic amino acid residues, Lys140, His172, His236, His237, Lys238, His241 and His251, lies in the vicinity of the 2-CMA binding site [Fig. 3(C)], implicating that the binding of substrate 2-CMA or MA to PcpE is mainly driven through ionic interactions. Except residue Lys140, the other six basic amino acid residues are strictly conserved in the maleylacetate reductase family. Specifically, residues His236, His237, Lys238 and His241 are located in a conserved region with amino sequence of ^235^LHHKLCHTLGG^245^, which could be considered as a signature motif for maleylacetate reductases. However, it is difficult to validate the accuracy of our docking result since the MA binding site has never been experimentally identified for any maleylacetate reductase. The putative 2-CMA/MA binding site for PcpE from our current docking study is located at the same site proposed for *Rm*-MR based on the binding site of a sulfate anion^30^, implying that PcpE model is likely to be reasonable to guide our subsequent mutagenesis and kinetics studies. Although other amino acid residues may also be involved in 2-CMA/MA binding, we were more interested in identifying whether any of the seven basic amino acid residues plays an essential role in the reductive reactions catalyzed by PcpE.

### Residues His172 and Lys238 are essential for the catalytic activity

To characterize the catalytic roles of the seven basic amino acid residues during PcpE catalysis, we mutated each of them into an alanine residue and measured the respective kinetic parameters (*K*_*m*_, *k*_*cat*_ and *k*_*cat*_/*K*_*m*_) using MA as the substrate and NADH as the co-substrate. The selection of MA instead of 2-CMA as the substrate in this kinetic study was due to three considerations: (1) the binding mode of MA to PcpE should be the same as that for 2-CMA; (2) the catalytic efficiency was previously reported to be very similar towards 2-CMA and MA for the maleylacetate reductase from *Pseudomonas* sp strain B13, which shares 51% sequence identity with PcpE^31^; and (3) we were not able to produce pure 2-CMA from its dienelactone precursor because of stability issues. As shown in Table 1, the catalytic activity was reduced for all mutated PcpE except for the H236A mutant.

**Table 1:** Apparent kinetic parameters for recombinant PcpE and its mutants.

Enzyme	*K*_*m*_ (mM)	*k*_*cat*_ (s^−1^)	*k*_*cat*_ */K*_*m*_ (s^−1^ mM^−1^)
Wild type	0.09 ± 0.04	1.2 ± 0.3	13.3
K140A	0.36 ± 0.40	0.9 ± 0.3	2.5
H236A	0.07 ± 0.04	2.1 ± 0.2	30.0
H237A	0.93 ± 0.18	3.8 ± 0.3	4.1
H241A	0.16 ± 0.08	1.7 ± 0.2	10.6
H251A	1.69 ± 0.89	8.8 ± 2.6	5.2

*No catalytic activity detected for the H172A and K238A mutants at final enzyme concentration of 200 µg/mL.

Compared to the wild type PcpE, the H236A mutant slightly decreased the apparent *K*_*m*_ from 0.09 ± 0.04 mM to 0.07 ± 0.04 mM, increased the apparent *k*_*cat*_ by about 1.8-fold from 1.2 ± 0.3 s^−1^ to 2.1 ± 0.2 s^−1^, and increased the apparent catalytic efficiency *k*_*cat*_ */K*_*m*_ by 2.3-fold from 13.3 s^−1^ mM^−1^ to 30.0 s^−1^ mM^−1^. This indicated that the H236A mutation mainly increases the substrate turnover for PcpE. Our calculation of the change in folding stability (ddG) showed that H236A mutation is favorable for the overall conformational stability of PcpE (Supplementary Table S1). In fact, the residue corresponding to His236 of PcpE is a small hydrophobic amino acid (such as alanine, valine and proline) in the alcohol dehydrogenases. This suggests that maleylacetate reductases might have sacrificed its conformational stability by mutating a small hydrophobic residue to a basic histidine residue in attempt to achieve binding affinity for the acidic substrates 2-CMA and MA during their molecular evolution from iron-containing alcohol dehydrogenases. Moreover, we speculated that the H236A mutation might also enhance the protonation of His237 by increasing its pKa values, which, in turn, promotes better substrate binding. Further studies are warranted to investigate how the H236A mutation elevates the substrate turnover of PcpE and whether mutation of His236 to hydrophobic residues other than alanine, such as valine, leucine, isoleucine, proline and phenylalanine, could result in an even greater increase in catalytic activity of PcpE.

Out of the other six mutants of PcpE, we did not detect any catalytic activity for the H172A and K238A mutants, indicating that they are essential for the catalytic activity of PcpE. Dynamic light scattering was performed to confirm that the mutations did not change the overall folding of PcpE. It has been reported in previous studies that histidine and lysine residues are actively involved in NAD+/NADH-dependent oxidoreductions^32,33^. It is warranted in future studies to identify what important roles His172 and Lys238 play in both substrate binding and electron transfer during the consecutive reduction reactions catalyzed by PcpE. The K140A mutant exhibited a 4-fold increase in *K*_*m*_ (0.36 ± 0.40 mM) and a slight decrease in *k*_*cat*_ (0.9 ± 0.3 s^−1^) compared to the wild type PcpE, resulting in a 5.3-fold decrease of the catalytic efficiency *k*_*cat*_/*K*_*m*_ from 13.3 s^−1^ mM^−1^ to 2.5 s^−1^ mM^−1^. As for the other three mutants of PcpE, H237A, H241A and H251A, both *K*_*m*_ and *k*_*cat*_ were augmented but with a reduction in the catalytic efficiency *k*_*cat*_/*K*_*m*_. The reduced catalytic efficiency for K140A, H237A, H241A and H251A mutants is likely due to decreased substrate binding resulted from the loss of ionic interactions between the positively charged side chains of these basic residues and the carboxylate groups of MA. Our results are also consistent with the enzymatic study on *Rm*-MR with the H243A mutant (corresponding to H241A mutant of PcpE), which exhibited reduced catalytic activity^30^. Further studies are warranted to identify whether mutation of these residues to other basic amino acids, such K140R, H237R, H251K and H251R, could improve the catalytic efficiency of PcpE.

### PcpE prefers NADH and 2-CMA

For bacterial catabolism, NADH rather than NADPH is normally preferred as the co-substrate^34^. However, NADPH-dependent alcohol dehydrogenases have also been identified^35,36^. Since PcpE, as well as the other catalytic enzymes in the PCP-biodegradation pathway of *S. chlorophenolicum* L-1, is still in the early stages of evolution, we undertook a kinetics study to understand its substrate preference in the consecutive reduction reactions with the apparent catalytic parameters summarized in Table 2. In this study, substrate 2-CMA was not purified from the preparative reaction mixture (containing ~70–80% 2-CMA) as it is stable for only 6–8 hours at room temperature. We first evaluated the substrate preference of PcpE for NADH or NADPH in the consecutive reduction reactions. Using 2-CMA as the co-substrate, PcpE exhibited apparent *K*_*m*_, *k*_*cat*_ and *k*_*cat*_/*K*_*m*_ of 0.13 ± 0.04 mM, 143.7 ± 15.3 s^−1^ and 1105.4 s^−1^ mM^−1^, respectively, towards NADH and 0.67 ± 0.75 mM, 29.7 ± 17.7 s^−1^ and 44.3 s^−1^ mM^−1^, respectively, towards NADPH, with an NADH/NADPH substrate specificity (*k*_*cat*_/*K*_*m*_ ratio) of 25.0. However, while using MA as the co-substrate, PcpE gave apparent *K*_*m*_, *k*_*cat*_ and *k*_*cat*_ */K*_*m*_ at 0.44 ± 0.57 mM, 1.2 ± 0.7 s^−1^ and 2.7 s^−1^ mM^−1^, respectively, towards NADH and 0.52 ± 0.37 mM, 0.7 ± 0.2 s^−1^ and 1.3 s^−1^ mM^−1^, respectively, towards NADPH, with an NADH/NADPH substrate specificity (*k*_*cat*_/*K*_*m*_ ratio) of only 2.1. These results suggest that PcpE favors NADH over NADPH as the co-substrate for its consecutive reduction reactions although the selectivity is much higher for the first reaction, reduction of 2-CMA to MA; and that the rate-limiting step for the catalytic function of PcpE is the reduction of MA to 3-OXO. Moreover, we examined PcpE for its substrate selectivity between 2-CMA and MA using NADH as the co-substrate. Also shown in Table 2, the apparent *K*_*m*_, *k*_*cat*_ and *k*_*cat*_ */K*_*m*_ were determined to be 0.09 ± 0.03 mM, 17.6 ± 1.8 s^−1^ and 195.6 s^−1^mM^−1^, respectively, towards 2-CMA and 0.09 ± 0.04 mM, 1.2 ± 0.3 s^−1^ and 13.3 s^−1^mM^−1^, respectively, towards MA. The 2-CMA/MA substrate specificity (*k*_*cat*_/*K*_*m*_ ratio) was 14.7 despite 2-CMA and MA exhibiting the same *K*_*m*_ towards PcpE. This further supported our above conclusion that the reduction of MA to 3-OXO was the rate-limiting step for PcpE catalysis.

**Table 2:** Apparent kinetic parameters for recombinant PcpE.

Co-substrate	Substrate	*K*_*m*_ (mM)	*k*_*cat*_ (s^−1^)	*k*_*cat*_ */K*_*m*_ (s^−1^mM^-1^)
2-CMA^*^	NADH	0.13 ± 0.04	143.7 ± 15.3	1105.4
	NADPH	0.67 ± 0.75	29.7 ± 17.7	44.3
MA	NADH	0.44 ± 0.57	1.2 ± 0.7	2.7
	NADPH	0.52 ± 0.37	0.7 ± 0.2	1.3
NADH	2-CMA^*^	0.09 ± 0.03	17.6 ± 1.8	195.6
	MA	0.09 ± 0.04	1.2 ± 0.3	13.3

*No purification.

**Figure 4:**
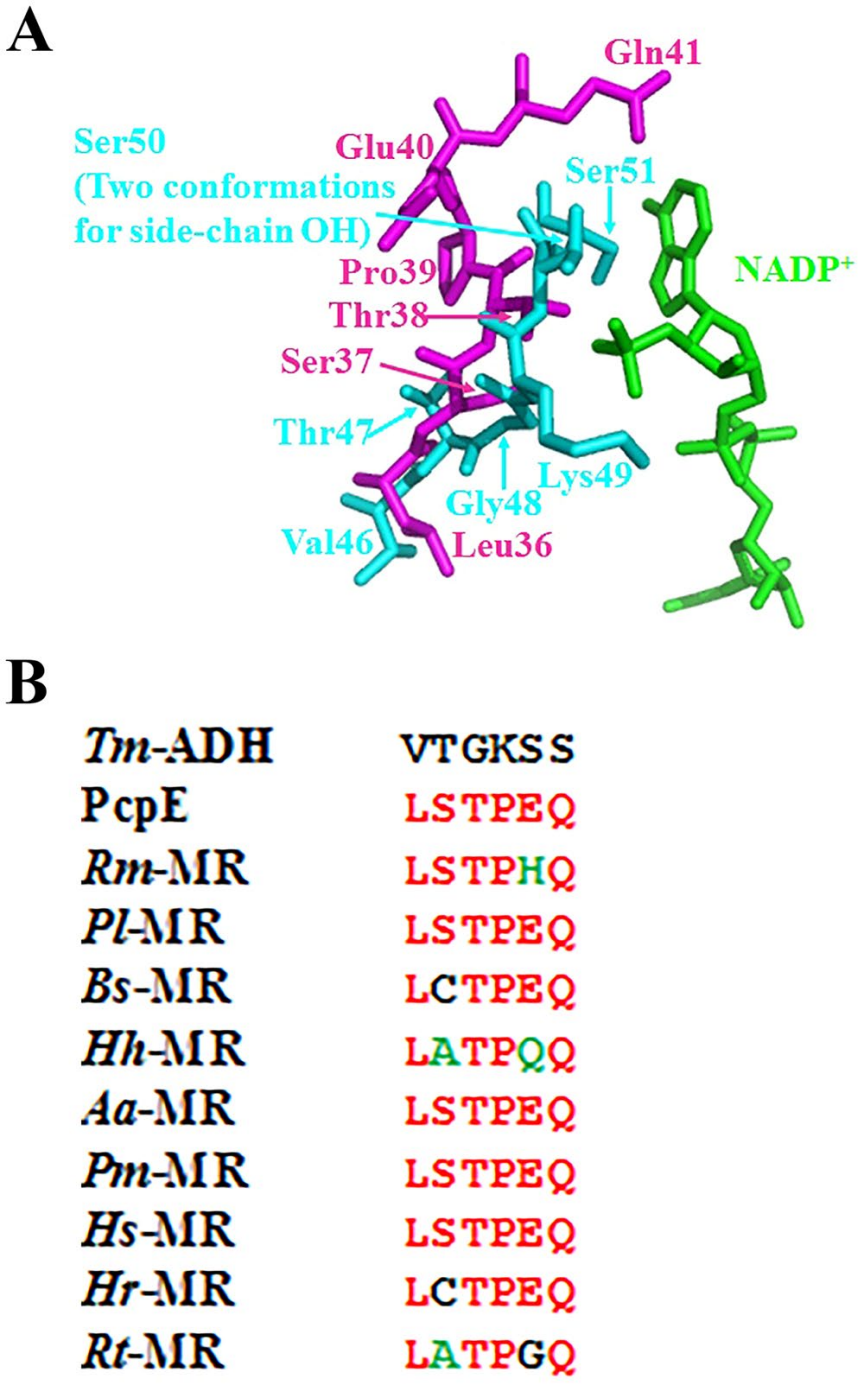
Maleylacetate reductase prefers NADH rather than NADPH as its co-substrate and the binding motif (amino acid sequence: LSTPEQ) for NADH is highly conserved in maleylacetate reductase. (**A**) Superimposition of PcpE model with the iron-containing alcohol dehydrogenase from *Thermotoga maritima* (*Tm-*ADH, PDB code: 1O2D) at the NADH/NADPH binding site with PcpE, *Tm*-ADH and NADP^+^ shown in cyan, magenta and green, respectively. (**B**) Multiple sequence alignment showing the conserved LSTPEQ motif in maleylacetate reductases. *Rm*-MR, *Pl*-MR, *Bs*-MR, *Hh*-MR, *Aa*-MR, *Pm*-MR, *Hs*-MR, *Hr*-MR and *Rt*-MR represent the maleylacetate reductase from *Rhizobium* sp. strain MTP-10005, *Pseudomonas* sp. Leaf127, *Burkholderia stabilis*, *Halomonas* sp. HAL1, *Azohydromonas australica*, *Paraburkholderia mimosarum*, *Halomonas salina*, *Hydrogenophaga* sp. RAC07 and *Ramlibacter tataouinensis*, respectively.

In order to get an insight on why PcpE favors NADH for its catalytic reactions, we compared our PcpE homology model with the crystal structure of *Tm*-ADH complexed with NADPH^29^. As shown in Fig. 4(A), the binding of the 2′-phosphate group of NADPH was mainly mediated *via* an ionic interaction with positively-charged Lys49 residue and hydrogen bonded with the polar Ser51 residue, with both residues located at the strand2-loop-helix3 (S2-loop-H3) region in the N-terminal α/β domain of *Tm*-ADH. In PcpE, the counterpart to the S2-loop-H3 region of *Tm*-ADH is a signature motif with the amino acid sequence LSTPEQ, which is highly conserved throughout maleylacetate reductases [Fig. 4(B)]. As a result of the loss of a positively-charged lysine residue and the introduction of a hydrophobic proline residue and a negatively-charged glutamate residue (as observed in PcpE), this signature motif would make it less favorable for NADPH to bind to the maleylacetate reductases. Furthermore, we examined the structure of the NADPH-dependent cinnamyl alcohol dehydrogenase from *Saccharomyces cerevisiae* (Scadh6p, PDB ID code: 1PIW) in order to get a glimpse of NADPH binding^37^. Interestingly, similar to our observation for *Tm*-ADH, the binding of the 2’-phosphate group of NADPH was mediated *via* ionic interactions with positively-charged residues Arg211 and Lys215 and hydrogen bonded with polar Ser210 residue, with all three residues located in the βB-loop-αC region of Scadh6p^37^. We subsequently performed a protein-protein BLAST (blastp) of the Scadh6p sequence against the non-redundant protein sequences (nr) database, and discovered that all three amino acid residues involved in binding the 2′-phosphate group of NADPH are conserved in NADPH-dependent alcohol dehydrogenases (data not shown). Thus, we concluded that NADPH-dependent alcohol dehydrogenases adopted basic residues (lysine and arginine) and a polar residue (serine) to elicit their substrate preference towards NADPH. Conversely, the NADH-dependent maleylacetate reductases, members of the iron-containing alcohol dehydrogenase superfamily, exert their substrate preference towards NADH over NADPH using the LSTPEQ signature motif to repulse the binding of NADPH.

### PcpE is inhibited by PCP in a concentration-dependent manner

PCP is a synthetic chemical rather than a natural product and was marketed as a low-cost and effective pesticide from the 1930s to the late 1980s, implicating that the PCP-biodegradation pathway in *S. chlorophenolicum* L-1 was assembled over a very short period of time and is still in the early stage of evolution. Because pentachlorophenol 4-monooxygenase (PcpB), the first and rate-limiting enzyme in the PCP-biodegradation pathway, possesses extremely low catalytic efficiency^20^, PCP may accumulate to higher concentrations inside the *S. chlorophenolicum* L-1 cells. Thus, in order to better understand how PCP is degraded by the bacterium, it is important to elucidate how the catalytic activities of the enzymes are affected in the presence of PCP.

**Figure 5:**
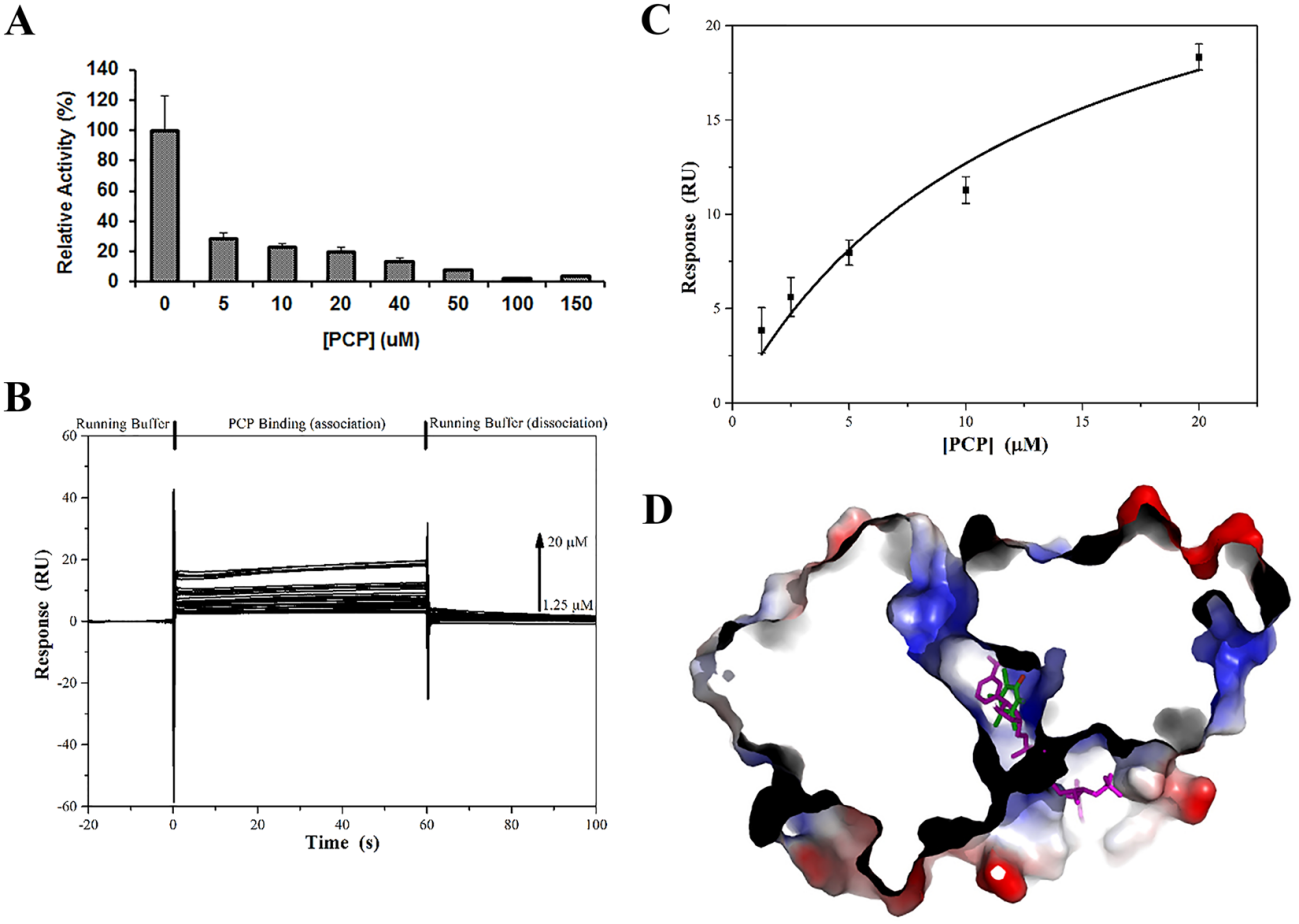
The inhibitory effect of PCP on PcpE. (**A**) Catalytic activity of PcpE is inhibited by PCP in a concentration-dependent manner. The reaction mixture (final volume: 240 µL) contained 20 mM Tris-HCl buffer, pH 7.0, 3 µg/mL PcpE, 360 µM NADH, 400 µM MA and PCP. The PCP concentrations used were 0, 5, 10, 20, 40, 50, 100 and 150 μM. A sample of PcpE boiled for 10 min was used as a blank control. The activity of PcpE (MA substrate) under different PCP concentrations was compared to the activity in the absence of PCP, which was assigned a relative activity of 100%. (**B**) Sensorgrams of concentration series of PCP flowing over a PcpE modified sensor surface. Contributions of bulk refractive index and non-specific binding were eliminated by subtracting the response of the PCP over an unmodified (control) surface. (**C**) The SPR response versus PCP concentration gives a dissociation constant (*K*_*d*_) of 12.8 ± 0.2 µM (n = 5). (**D**) Global docking study (grid box encompassed the whole PcpE model) showed that most favorable docking site for PCP was located at the site that binds the 5-(3-carbamoyl-1(4 H)-pyridinyl)-3,4-dihydroxytetrahydro-2-furanyl part of NADH or NADPH.

It is intriguing to identify how accumulated PCP could affect the catalytic efficiency of the PCP-biodegradation pathway in *S. chlorophenolicum* L-1. Previous studies showed that PCP may exert opposite effects on the catalytic enzymes^25,38^. On one hand, it induces the expression of genes *pcpE*, *pcpA* and *pcpR* ^25^. For example, the expression of *pcpE* was increased by almost 14-fold upon induction with 75 µM PCP^25^. On the other hand, PCP was shown to inhibit the enzymatic activity of tetrachlorobenzoquinone reductase (PcpD)^38^. We also speculated that PCP might inhibit other catalytic enzymes in the PCP-biodegradation pathway, such as the tetrachlorohydroquinone dehalogenase (PcpC), since it is severely inhibited by its chloroaromatic substrates tetrachlorohydroquinone and trichlorohydroquinone (both bearing high structural similarities to PCP)^39^. To evaluate whether PCP could impose any effect on PcpE, other than inducing its gene expression, we measured the catalytic activity of PcpE in the presence of 5–150 µM PCP [Fig. 5(A)]. Surprisingly, PcpE was inhibited by PCP in a concentration-dependent manner. At 5 µM PCP, PcpE maintained about 30% of catalytic activity; whereas as the PCP concentration reached 100 µM, less than 5% of catalytic activity remained for PcpE. Together with our previous results showing that the original deposited strain of *S. chlorophenolicum* ATCC39723 is no longer able to degrade PCP due to lack of functional PcpE^26^, the current study strongly suggests that PcpE serves as another chokepoint other than PcpB in the PCP-biodegradation pathway, and *S. chlorophenolicum* L-1 may function more efficiently when the concentration of PCP is low. In addition, our findings reinforced previous studies by Cai *et al*. that a second maleylacetate reductase might be involved in PCP biodegradation in *S. chlorophenolicum* L-1^25^.

It is not to our surprise that PCP is capable of inhibiting PcpD and possibly PcpC. PCP is a chloroaromatic compound and structurally resembles tetrachchlorobenzoquinone (TCBQ) and tetrachlorohydroquinone (TCHQ), and thus may act a competitive inhibitor of PcpD and PcpC. However, PCP does not bear any structural similarity with either 2-CMA or MA except the negative charge on the hydroxyl group (in aqueous environment), as it is aromatic and hydrophobic whereas 2-CMA and MA are aliphatic and hydrophilic. Because of the high hydrophobicity of the pentachlorophenyl moiety, PCP is less favorable than 2-CMA/MA to bind to the highly positively charged substrate binding site of PcpE. Hence, important questions that needed to be addressed were whether PCP binds to PcpE directly and where PCP-PcpE binding would occur. To tackle these questions, we first undertook a binding study using surface plasmon resonance (SPR). As shown in Fig. 5(B,C), PCP bound to PcpE with a dissociation constant *K*_*d*_ of 12.8 ± 0.2 µM (n = 5), suggesting to us that the binding of PCP to PcpE was reasonably strong. Subsequently, we searched for the binding site of PCP using the molecular docking method with a grid box encompassing the whole PcpE model. Remarkably, we discovered that PCP was predominantly docked into the positively charged solvent channel where the putative active site is located, with the best docking site located at the 5-(3-carbamoyl-1(4 H)-pyridinyl)-3,4-dihydroxytetrahydro-2-furanyl part of NADH or NADPH [Fig. 5(D)]. Our docking result is consistent with previous studies that aromatic groups can bind to cationic amino acid residues^40^. This implied that PCP might act as a competitive inhibitor of PcpE *via* binding to the NADH-binding site. However, we could not eliminate the possibility that PCP binds to the 2-CMA/MA binding site or other sites in the positively charged solvent channel *via* ionic interactions. It is warranted in the future studies to confirm whether the loss of catalytic activity for PcpE in the presence of PCP is due to binding to the NADH site, 2-CMA/MA site, both NADH and 2-CMA/MA sites, or other parts in the vicinity of the active site and identify the type of inhibition on PcpE imposed by PCP.

### PcpE possesses extremely low but detectable alcohol dehydrogenase activity towards ethanol

**Figure 6:**
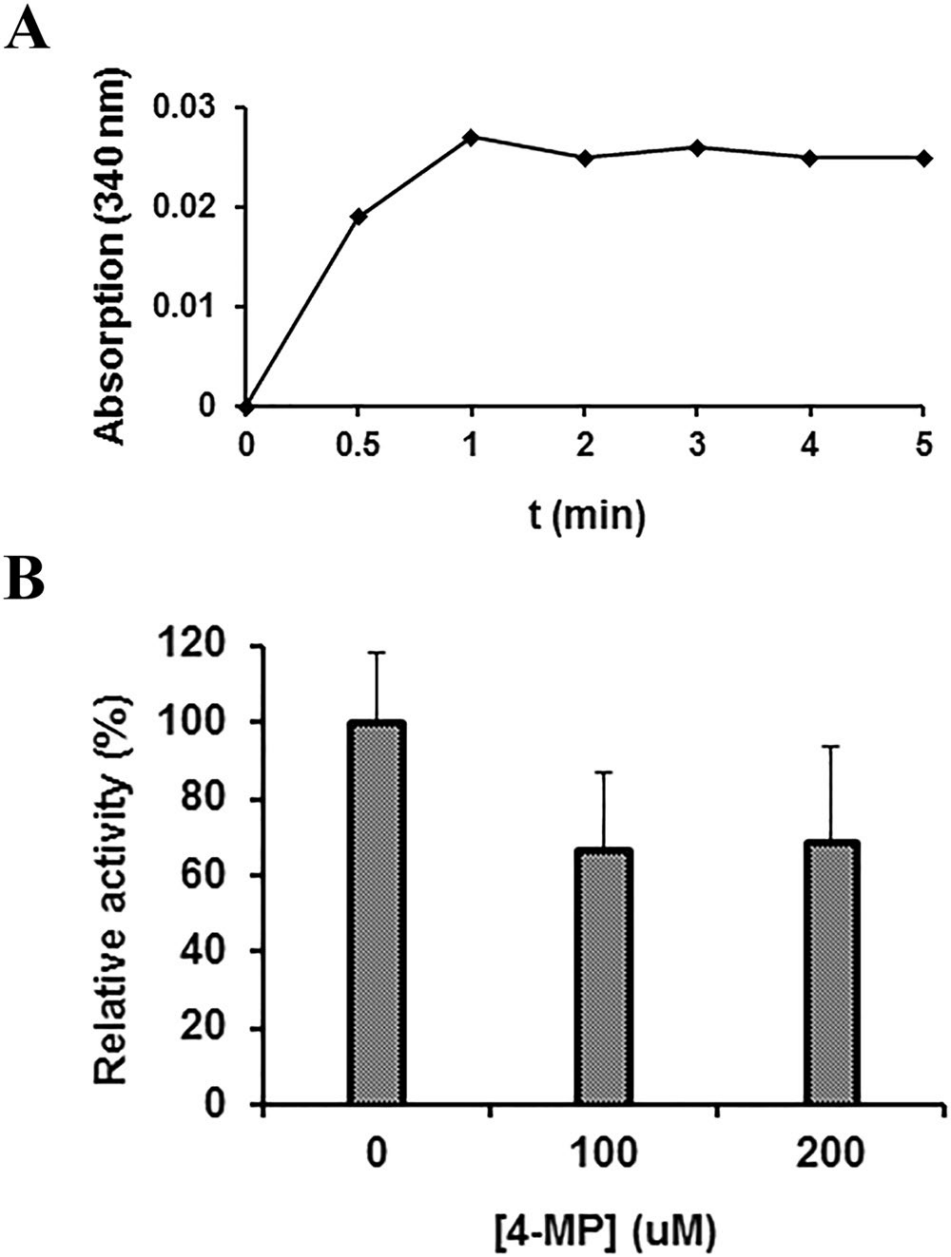
Alcohol dehydrogenase activity of PcpE towards ethanol (**A**) and inhibition of the reductase activity of PcpE by 4-methylpyrazole (**B**). (**A**) The alcohol dehydrogenase activity of PcpE was evaluated by monitoring the generation of NADH at 30 sec, 1 min, 2, min, 3 min, 4 min and 5 min, with the reaction mixture (final volume: 1 mL) contained 20 mM PBS buffer, pH 8.0, 200 µM ethanol, 500 µM NAD^+^ and 30 µg/mL PcpE. A sample of PcpE boiled for 10 min was used a blank control. (**B**) The inhibitory effect of 4-MP on PcpE was evaluated by monitoring the loss of NADH at 3 min after initiation of the reaction. The reaction system (final volume: 240 µL) contained 20 mM Tris-HCl buffer, pH 7.0, 3 µg/mL PcpE, 360 µM NADH, 400 µM MA and 4-MP. The 4-MP concentrations were 0, 100 and 200 μM, respectively. A sample of PcpE boiled for 10 min was used a blank control. The activity of PcpE under different 4-MP concentrations was compared to the activity in the absence of 4-MP, which was assigned a relative activity of 100%.

PcpE is an NADH-dependent reductase likely evolved from an iron-containing alcohol dehydrogenase. As the PCP-biodegradation pathway in *S. chlorophenolicum* L-1 was assembled over a very short period of time and the catalytic enzymes are in the early stage of molecular evolution, we would like to investigate how far PcpE has evolved from the alcohol dehydrogenases and whether it still possesses any alcohol dehydrogenase activity. Some bacterial iron-containing alcohol dehydrogenases have exhibited catalytic activity towards ethanol^41,42^. Thus, we decided to evaluate whether PcpE could exhibit any dehydrogenase activity towards ethanol using boiled PcpE sample as a negative control. No alcohol dehydrogenase activity was detected for the boiled PcpE sample. However, PcpE exhibited very low but measurable catalytic activity towards ethanol, and conversion of ethanol to acetaldehyde reached an equilibrium within 1 min [Fig. 6(A)]. The final concentration of NADH in the reaction mixture was approximately 4.3 µM, which corresponded to an ethanol-to-acetaldehyde conversion rate of less than 2.2% under the initial experimental condition. This result supports the notion that PcpE is in the early stage of molecular evolution from an alcohol dehydrogenase to a reductase and possesses low levels of alcohol dehydrogenase activity. We did not investigate whether PcpE would exhibit higher alcohol dehydrogenase activity towards other alcohols. Furthermore, we examined whether the reductase activity of PcpE could be affected by 4-methylpyrazole (4-MP) as it is a competitive inhibitor of ethanol and has a stronger binding towards alcohol dehydrogenases^43^. As shown in Fig. 6(B), the reductase activity of PcpE was reduced by about 33% (p = 0.10) in the presence of 100 µM of 4-MP and 31% (P = 0.11) in the presence of 200 µM of 4-MP, respectively. However, the decrease of reductase activity is not statistically significant at either concentration of 4-MP. In addition, the inhibition of PcpE by 4-MP is not concentration-dependent, suggesting that binding of 4-MP to PcpE is weak and 4-MP may achieve its maximum inhibitory effect on PcpE at concentration lower than 100 µM. From these studies, we concluded that PcpE is indeed evolved in the early stage from an iron-containing alcohol dehydrogenase with its substrate binding site adapted from binding of a neural alcohol molecule to binding of the acidic 2-CMA and MA molecules.

## Conclusion

In conclusion, we investigated the enzyme PcpE using site-directed mutagenesis, enzyme kinetics, surface plasmon resonance and *in silico* techniques. We determined that His172 and Lys238 are essential for PcpE activity, the H236A mutant increases the catalytic efficiency and PcpE preferentially binds NADH to NADPH which is consistent with the lack of positively-charged lysine residue and presence of negatively-charged glutamate residue in the active site similar to that observed in NADH-dependent maleylacetate reductases. In addition, we determined that PCP is a concentration-dependent inhibitor of PcpE. Together, these results indicate that further evolution to more catalytically active PcpE may be an important contributor to improved *S. chlorophenolicum* L-1-mediated bioremediation of PCP.

## Materials and Methods

### Materials

All chemicals except 2,6-dichlorohydroquinone (DCHQ) were purchased from Sigma-Aldrich Canada (Oakville, ON, Canada). DCHQ was purchased from 3B Scientific Corporation (Libertyville, IL, USA). The synthesis of the dienelactone precursor of MA, (E)-5-oxo-2,5-dihydrofuran-2-ylideneacetic acid, was reported previously^26^. The overexpression and purification of recombinant dichlorohydroquinone dioxygenase (PcpA) was also reported previously^44^. The preparation of 2-CMA was carried out in a quartz cuvette with the reaction mixture (final volume: 1 mL) containing oxygen-saturated 20 mM Tris-HCl buffer, pH 7.0, 100 µg/mL PcpA, 5 mM DCHQ and 100 µM ascorbic acid. The reaction was allowed to proceed in darkness for 2 hr at room temperature (~23 °C) before being terminated by removing PcpA from the reaction mixture using an Amicon^®^ 10 kDa cutoff filter. The yield of 2-CMA was about 70–80% as calculated by the decrease of UV absorption for DCHQ at 305 nm. The substrate 2-CMA, containing residual DCHQ and ascorbic acid, was not further purified for the enzymatic studies of PcpE as it is stable only for 6–8 hr at room temperature. UV-transparent 96-well plates used for the steady-state kinetic studies of PcpE and its mutants were purchased from VWR International (Mississauga, ON, Canada).

### PcpE structural model and docking of 2-CMA, NADH and PCP

The structural model of PcpE was constructed using a homology/comparative modeling method. The modeling template, an iron-containing alcohol dehydrogenase from *Thermotoga maritima* (*Tm-ADH*, PDB ID: 1O2D), was identified from a protein-protein BLAST (blastp) search against the Protein Data Bank (PDB) using the amino acid sequence of PcpE. Pair-wise comparative sequence alignment between PcpE and *Tm-ADH* was performed by ClustalW^45^. Ten initial models of PcpE were built using the program MODELLER version 8.1 with default parameters^46^. The geometry and energy criteria for each initial model were subsequently evaluated using programs PROCHECK^47^, ProSa II^48^ and Verify3D^49^. The model with lowest energy and best geometry was chosen as the final structural model for PcpE. The docking of 2-CMA, NADH or PCP (anion form) to PcpE was performed with AutoDock3^50^. For 2-CMA and NADH, the docking grid box encompassed only the putative active site, which is located within a positively-charged solvent channel; whereas for PCP, the docking grid box encompassed the whole PcpE model. For each molecule, the top 50 out of 100 initial docking solutions were selected for subsequent energy minimization using the *Monte Carlo* simulated annealing feature of Autodock3^50^, and the best solution after energy minimization was designated as the final docked conformation.

### Cloning, overexpression and purification of PcpE and its mutants

The cloning, overexpression and purification of recombinant PcpE was reported previously^26^. PcpE point mutants were prepared using overlap extension PCR method (Molecular Cloning, A Laboratory Manual, 3^rd^ ed., Cold Spring Harbor Laboratory Press, 2001). *Nde*I and *Xho*I restriction sites were introduced in the forward (*pcpE*F) and reverse (*pcpE*R) end primers, respectively^26^. For each point mutation of PcpE, a pair of mutagenic primers (EMF and EMR) containing the mutation was designed to be completely complementary to each other (Supplementary Table S2). In the first round of PCR, two separate PCR reactions were set up using one of the end primers and the matching mutagenic primers, *i.e. pcpE*F + EMRs and *pcpE*R + EMFs. The two DNA fragments obtained from the first round of PCR were gel purified and then used for a second round of PCR including both end primers to get the full length of *PcpE* gene with the mutations. The mutated DNA fragments obtained from the second round of PCR were gel purified and subcloned into the protein expression vector pET30a(+) at the *Nde*I and *Xho*I sites. The resulting plasmid pET30a(+)-*pcpEM* for each PcpE mutant was subsequently transformed into *Escherichia coli* DH5α cells. Positive transformants on the LB-agar culture plates containing 30 mg/mL kanamycin were confirmed by DNA sequencing. The recombinant plasmid pET30a(+)-*pcpEM* extracted from the positive DH5α clones was transformed into *E. coli* BL21-AI cells for overexpression of the His_6_-tagged recombinant PcpE mutants. The PcpE mutants were purified using the same protocol for PcpE^26^.

### Steady-state kinetic studies of PcpE and its mutants

All experiments in the current study were carried out in triplicate. The catalytic activity of PcpE and its mutants was assayed using a protocol previously reported^26^. Briefly, the catalytic activity of PcpE and its mutants towards MA or 2-CMA was assayed by monitoring the loss of UV absorption for NADH at 340 nm at 10 sec intervals over a period of 8 min at room temperature (~23 °C) using UV-transparent 96-well plates. For the substrate MA, the reaction (final volume: 240 µL) was carried out in 20 mM Tris-HCl buffer, pH 7.0, 360 µM NADH and MA (final concentration: 50 µM – 2 mM). The amount of PcpE or its mutants added to the reaction mixture varied with the final concentration ranging from 3 µg/mL to 200 µg/mL based on a pilot study. No catalytic activity was detected for the H172A and K238A mutants even at the final enzyme concentration of 200 µg/mL. For the substrate 2-CMA, the reaction system (final volume: 240 µL) contained 20 mM Tris-HCl buffer, pH 7.0, PcpE 1.5 µg/mL, 360 µM NADH and 2-CMA (final concentration: 50 µM – 1 mM). A sample of PcpE boiled for 10 min was used as a blank control. Furthermore, adding 25 µM ascorbic acid and 50 µM DCHQ to the reaction system using MA as the substrate did not significantly change the catalytic activity of PcpE. The apparent *K*_*m*_ and *k*_*cat*_ values were determined by nonlinear regression analysis (initial reaction rate versus substrate concentration) using GraphPad Prism 3.03 software (GraphPad Software, San Diego, CA, USA). The extinction coefficient of 6.22 mM^−1^ cm^−1^ for both NADH and NADPH and the path length of the absorption of 0.71 cm were used in the calculations.

### Substrate preference of PcpE towards NADH/NADPH

The preference of PcpE towards its co-substrate NADH/NADPH was also evaluated by monitoring the loss of UV absorption for NADH or NADPH at 340 nm at 10 sec intervals over a period of 8 min at room temperature (~23 °C) using UV-transparent 96-well plates. For MA, the reaction system (final volume: 240 µL) contained 20 mM Tris-HCl buffer, pH 7.0, 3 µg/mL PcpE, 300 µM MA, and NADH (final concentration: 50 µM – 1 mM) or NADPH (final concentration: 50 µM – 1.8 mM); whereas for 2-CMA, the reaction system (final volume: 240 µL) contained 20 mM Tris-HCl buffer, pH 7.0, 0.2 µg/mL PcpE, 250 µM 2-CMA, and NADH (final concentration: 50 µM – 1 mM) or NADPH (final concentration: 50 µM – 1.8 mM). A sample of PcpE boiled for 10 min was used as a blank control. The apparent *K*_*m*_ and *k*_*cat*_ values were determined by nonlinear regression analysis (initial reaction rate versus substrate concentration) using GraphPad Prism 3.03 software.

### Effect of PCP and 4-methylpyrazole (4-MP) on the reductase activity of PcpE

The effects of PCP and 4-MP on the reductase activity of PcpE were determined using the same method described above in the section for *steady-state kinetic studies of PcpE and its mutants* with the reaction system (final volume: 240 µL) containing 20 mM Tris-HCl buffer, pH 7.0, 3 µg/mL PcpE, 360 µM NADH and 400 µM MA. To determine the effect of PCP, it was added to the reaction system to achieve final concentrations of 5, 10, 20, 40, 50, 100 and 150 μM. To determine the effect of 4-MP, it was added to the reaction system to achieve final concentrations of 100 µM and 200 µM. The enzymatic reaction was allowed to proceed for 3 min and UV absorption monitored at 340 nm. The decrease of UV absorption for the reaction system in the absence of PCP and 4-MP was used as the standard in the relative activity comparisons.

### Alcohol dehydrogenase activity of PcpE

The alcohol dehydrogenase activity of PcpE was evaluated by monitoring the generation of NADH at 30 sec, 1 min, 2, min, 3 min, 4 min and 5 min after initiation of the reaction. The reaction system (final volume: 1 mL) contained 20 mM PBS buffer, pH 8.0, 200 µM ethanol, 500 µM NAD^+^ and 30 µg/mL PcpE. UV absorption at 340 nm was obtained using a UV-Vis spectrophotometer from Thermo Fisher Scientific-Canada (Burlington, ON, Canada). A sample of PcpE boiled for 10 min was used as a blank control.

### Surface plasmon resonance (SPR) study of PCP binding to PcpE

SPR data on the binding of PCP to PcpE were collected using a Bio-Rad ProteOn™ XPR36 Protein Interaction Array and analyzed using the Bio-Rad ProteOn™ Manager V3.0.5 at the Saskatchewan Structural Sciences Centre (Saskatoon, SK, Canada). Briefly, His_6_-tagged PcpE was covalently immobilized onto a ProteOn™ GLH flow channel in the vertical direction by amine coupling using filtered 1X PBS-T running buffer (10 mM phosphate, 150 mM NaCl, 0.05% Tween-20 and 5% DMSO, pH 7.4). The GLH flow channel was activated with a 1:1 (v/v) aqueous solution of 1-ethyl-3-(3-dimethylaminopropyl) carbodiimide hydrochloride (EDAC, 20 mM) and sulfo-NHS (N-hydroxysulfosuccinimide) (5 mM) for 5 min (30 µL/min). The activation was immediately followed by a 5-min injection of PcpE (56 µg/mL in 10 mM acetate buffer, pH 5.0), which also contained 250 µM PCP, and a final solution concentration of 5% DMSO. The addition of PCP helped to protect the enzyme from immobilizing onto the sensor chip *via* lysine residues in the active binding site. The immobilization was followed up with a 5-min injection of 1 M ethanolamine (30 µL/min) in order to deactivate any remaining active sites. The data were corrected for DMSO interaction with the reference surface using the EDC calibration function in the software. The association time and the dissociation time were 60 sec and 40 sec, respectively.
